# Oleanolic Acid Acetate Alleviates Cisplatin-Induced Nephrotoxicity via Inhibition of Apoptosis and Necroptosis In Vitro and In Vivo

**DOI:** 10.3390/toxics12040301

**Published:** 2024-04-18

**Authors:** Bori Lee, Yeon-Yong Kim, Seungwon Jeong, Seung Woong Lee, Seung-Jae Lee, Mun-Chual Rho, Sang-Hyun Kim, Soyoung Lee

**Affiliations:** 1 Functional Biomaterials Research Center, Korea Research Institute of Bioscience and Biotechnology (KRIBB), Jeongeup 56212, Republic of Korea; leebori1004@kribb.re.kr (B.L.); gyy123@kribb.re.kr (Y.-Y.K.); jsw0212@kribb.re.kr (S.J.); lswdoc@kribb.re.kr (S.W.L.); seung99@kribb.re.kr (S.-J.L.); rho-m@kribb.re.kr (M.-C.R.); 2 Cell and Matrix Research Institute, Department of Pharmacology, School of Medicine, Kyungpook National University, Daegu 41944, Republic of Korea

**Keywords:** acute kidney injury, oleanolic acid acetate, cisplatin, necroptosis, apoptosis

## Abstract

Cisplatin is a widely used anti-cancer drug for treating solid tumors, but it is associated with severe side effects, including nephrotoxicity. Various studies have suggested that the nephrotoxicity of cisplatin could be overcome; nonetheless, an effective adjuvant drug has not yet been established. Oleanolic acid acetate (OAA), a triterpenoid isolated from *Vigna angularis*, is commonly used to treat inflammatory and allergic diseases. This study aimed to investigate the protective effects of OAA against cisplatin-induced apoptosis and necroptosis using TCMK-1 cells and a mouse model. In cisplatin-treated TCMK-1 cells, OAA treatment significantly reduced Bax and cleaved-caspase3 expression, whereas it increased Bcl-2 expression. Moreover, in a cisplatin-induced kidney injury mouse model, OAA treatment alleviated weight loss in the body and major organs and also relieved cisplatin-induced nephrotoxicity symptoms. RNA sequencing analysis of kidney tissues identified lipocalin-2 as the most upregulated gene by cisplatin. Additionally, necroptosis-related genes such as receptor-interacting protein kinase (RIPK) and mixed lineage kinase domain-like (MLKL) were identified. In an in vitro study, the phosphorylation of RIPKs and MLKL was reduced by OAA pretreatment in both cisplatin-treated cells and cells boosted via co-treatment with z-VAD-FMK. In conclusion, OAA could protect the kidney from cisplatin-induced nephrotoxicity and may serve as an anti-cancer adjuvant.

## 1. Introduction

Cisplatin, a platinum-based agent, is one of the most widely used drugs in the treatment of solid tumors such as lung, ovarian, and testicular tumors. However, cisplatin is known to induce organ toxicity, particularly nephrotoxicity [1]. Previous reports identified kidney toxicity in approximately 30% of cancer patients treated with cisplatin [2]. These studies demonstrated that cisplatin treatment significantly increased the reactive oxygen species, blood urea nitrogen (BUN), and serum creatinine levels in the kidney injury model [3,4]. Nephrotoxicity was accompanied by rapid renal dysfunction along with electrolytes and hematologic abnormalities, fluid overload, and multi-organ failure [5]. Even small changes in kidney function can lead to complications [6]. An increase in serum creatinine levels has been associated with higher mortality and longer hospital stays in patients with acute renal failure [7]. Additionally, BUN is often used as a clinical index for kidney injury and function, similar to creatinine [8]. Furthermore, lipocalin-2 increased in urine before significant changes in clinical chemistry parameters were evident in kidney injury and acute renal failure. These studies demonstrate the potential of LCN2 as a biomarker of acute kidney injury in various disease processes [9,10,11].

Cisplatin is a DNA-damaging agent that interferes with DNA replication [12], inducing programmed kidney cell death such as apoptosis, necroptosis, and ferroptosis [13,14]. An important mechanism of cisplatin-induced DNA damage in cell death is apoptosis [15], which is a form of programmed cell death occurring in multi-cellular organisms. In the apoptotic process, Bcl-2 (an anti-apoptotic protein) antagonizes Bax (a pro-apoptotic protein), triggering the activation of caspases [16,17]. Bcl-2 prevents the release of cytochrome C from the mitochondria, whereas Bax induces the release of cytochrome C [18]. Cytochrome C activates caspase-9, subsequently inducing the cleavage of caspase-3 and poly ADP-ribose polymerase (PARP) [19,20]. Unlike necrosis, apoptosis involves the removal of apoptotic bodies by phagocytes, and cellular contents do not spill out into other cells [21].

In contrast to programmed cell death mechanisms such as apoptosis, necroptosis (also known as programmed necrosis) is initiated by cellular damage or pathogens [22,23] and is primarily triggered by tumor necrosis factor-α (TNF-α) receptor-mediated necroptosis-related proteins [24,25,26]. Under certain conditions, TNF receptor activation leads to the phosphorylation of receptor-interacting protein kinase 1 (RIPK1), recruiting RIPK3. The RIPK1/RIPK3 complex then recruits and phosphorylates the mixed lineage kinase domain-like protein (MLKL). Ultimately, the phosphorylation of MLKL by RIPK3 results in necroptosis via plasma membrane disruption and cell lysis [25,27,28,29,30]. Additionally, depending on the cell type and context, the activation of RIPK1 causes apoptosis or inflammation [31,32]. Furthermore, necrostatin-1 (Nec-1) can block necroptosis by inhibiting the phosphorylation of RIPK1 [33]. The z-VAD-FMK can hamper the activation of caspase-3, thereby blocking apoptosis and boosting necroptosis [34].

Therefore, the search for new adjuvants is crucial to mitigate cisplatin-induced kidney toxicity via cell death. Oleanolic acid acetate (OAA), a triterpenoid compound isolated from *Vigna angularis*, is commonly used in daily diet or as a traditional medicine in Asia [35,36]. Previous studies have demonstrated the various pharmacological activities of *V. angularis* and OAA, such as anti-osteoporotic, anti-inflammatory, and anti-allergic activities [35,37]. Nevertheless, the effects of OAA on cisplatin-induced nephrotoxicity have not yet been investigated.

In the present study, we focused on the inhibitory effects of OAA on cisplatin-induced cell death, particularly apoptosis and necroptosis caused by cisplatin treatment. Consequently, we evaluated the protective effects and mechanisms of action of OAA against cisplatin-induced nephrotoxicity in the mouse kidney cell line TCMK-1 and a mouse kidney injury model.

## 2. Materials and Methods

### 2.1. Materials

Cisplatin, sodium thiosulfate (ST), oleanolic acid (OA), necrostatin-1 (Nec-1), and z-VAD-FMK were purchased from Sigma-Aldrich (St. Louis, MO, USA). OAA was purified from *V. angularis* as previously described [36]. Briefly, the *V. angularis* material was dried, pulverized to a fine powder, and extracted twice with 95% ethanol (EtOH) at 70 °C. The EtOH extracts were concentrated under reduced pressure. For fractionation, the EtOH extract was resuspended in water and then extracted with ethyl acetate (EtOAc). To isolate compound from the EtOAc fraction, it was further chromatographed on silica gel using a gradient hexane-EtOAc solvent system. The recrystallization of H3 in EtOAc yielded OAA. The primary antibodies RIP (#3493, rabbit monoclonal), phospho-RIP (#65746, rabbit monoclonal), RIP3 (#95702, rabbit monoclonal), phospho-RIP3 (#91702, rabbit monoclonal), MLKL (#37705, rabbit monoclonal), phospho-MLKL (#37333, rabbit monoclonal), and β-actin (#4967S, rabbit monoclonal), as well as the anti-rabbit IgG horseradish peroxidase-conjugated secondary antibody (#7074S) and anti-mouse IgG horseradish peroxidase-conjugated secondary antibody (#7076S), were purchased from Cell Signaling Technology (Danvers, MA, USA).

### 2.2. Cell Culture

The mouse epithelial kidney cell line TCMK-1 (CCL-139) was purchased from the American Type Culture Collection (Manassas, VA, USA). Cells were maintained in Eagle’s minimum essential medium (EMEM) supplemented with heat-inactivated 10% fetal bovine serum, 100 U/mL of penicillin G, and 100 μg/mL of streptomycin at 37 °C in 5% CO_2_.

### 2.3. Cell Viability

The cellular toxicity of OAA in TCMK-1 cells was analyzed using an EZ-Cytox assay kit (Dogen, Seoul, Republic of Korea) according to the manufacturer’s protocol. Briefly, TCMK-1 cells (1 × 10^5^ cells/well in a 96-well plate) were seeded with 100 μL of EMEM for 24 h. The cells were treated with various OAA concentrations for 21 h, and 50 μL of supernatant was transferred to a new 96-well plate. The supernatant was mixed with 50 μL of water-soluble tetrazolium salt (WST-1) solution, and absorbance was then measured at 450 nm using a microplate reader (Thermo Fisher Scientific, Waltham, MA, USA). Cell viability was calculated as the relative absorbance compared to control. The experiment was repeated three times.

### 2.4. Flow Cytometric Analysis

The apoptosis assay was performed using the FITC Annexin V apoptosis detection kit (556547; BD Biosciences, Oxford, UK). TCMK-1 cells (2 × 10^5^ cells/well in a 12-well plate) were cultured in 1 mL of EMEM for 24 h. Cells were treated with 1000 µM of sodium thiosulfate and various OAA concentrations for 1 h and subsequently stimulated with 20 µM of cisplatin. Sodium thiosulfate, a chelator of cisplatin, was used as a positive control drug [38]. After 21 h, the cells were harvested and stained with FITC Annexin V and propidium iodide. Stained cells were subjected to flow cytometry using a BD Accuri C6 Plus flow cytometer (BD Biosciences). The gate strategy for the total cells was counted as 20,000, and the Annexin V/propidium iodide ratio was determined. The concentration of each drug was used as previously described. [39,40]. The experiment was repeated three times.

### 2.5. Proteome Profiler Mouse Apoptosis Array

The expression of apoptosis-related proteins was screened using a proteome profiler mouse apoptosis array (R&D Systems, Minneapolis, MN, USA) according to the manufacturer’s protocol. Briefly, TCMK-1 cells (2 × 10^5^ cells/well in a 6-well plate) were cultured in 2 mL of EMEM for 24 h. The cells were treated with 1000 µM of sodium thiosulfate and 30 µM of OAA for 1 h and subsequently stimulated with 20 µM of cisplatin. After 21 h, the cells were harvested and lysed in ice-cold cell lysis buffer (Cell Signaling Technology) containing phosphatase and protease inhibitor cocktail (0.5 mM PMSG/DTT and 5 μg/mL leupeptin/aprotinin) for 30 min at 4 °C. Cell lysates were incubated with nitrocellulose membranes containing antibodies against apoptosis-related proteins. Signals were visualized using a chemiluminescent substrate (Thermo Scientific, Waltham, MA, USA) and detected by the ChemiDoc XRS imaging system (Bio-Rad Laboratories, Hercules, CA, USA). The experiment was repeated three times.

### 2.6. Animals

All 8-week-old C57BL/6 mice were purchased from Orient Bio (Gwangju, South Korea) and housed in a controlled environment with constant humidity (55 ± 5%) and temperature (22 ± 2 °C) under a 12 h dark/12 h light cycle, with a standard laboratory diet and water supply. The care and treatment of animals were conducted in accordance with the guidelines established by the Public Health Service Policy on the Humane Care and Use of Laboratory Animals and were approved by the Institutional Animal Care and Use Committee of the Korea Research Institute of Bioscience and Biotechnology (approval no.: KRIBB-AEC-21054; date of approval: 22 February 2021).

### 2.7. Mouse Model of Cisplatin-Induced Nephrotoxicity

The mice were randomly divided into the following six experimental groups, with seven mice each: (1) control group; (2) group treated with 20 mg/kg cisplatin; (3) group treated with 20 mg/kg cisplatin and 1 g/kg sodium thiosulfate; (4) group treated with 20 mg/kg cisplatin and 50 mg/kg OA; (5) group treated with 20 mg/kg cisplatin and 25 mg/kg OAA; and (6) group treated with 20 mg/kg cisplatin and 50 mg/kg OAA. OA and OAA were dissolved in a 0.5% carboxymethylcellulose solution and orally administered once daily for 5 days. ST was dissolved in 0.9% saline and intraperitoneally injected once daily for 5 days. Cisplatin was dissolved in 0.9% saline and intraperitoneally injected as a single dose (20 mg/kg) 1 h after drug administration on the first day. The dose of each drug was used as previously described [37,41,42].

### 2.8. Serum and Tissue Collection

At 24 h after the last cisplatin injection, the mice were sacrificed via 4% isoflurane exposure. Blood samples were obtained from the mouse hearts, and the collected blood samples were held at room temperature for 3 h. Subsequently, serum was obtained via centrifugation at 3000 rpm for 15 min at 4 °C. Organs (kidneys, liver, and spleen) were harvested and washed with cold saline. The weight of the organs was measured, and the kidneys were then fixed in 4% formaldehyde solution at room temperature for histopathology. Serum and organ samples were stored at −80 °C until use.

### 2.9. Serum Analysis

Serum biochemical levels of BUN and creatinine were measured using FUJIFILM DRI-CHEM NX500 with DRI-CHEM slide (FUJIFILM, Tokyo, Japan) according to the manufacturer’s protocol.

### 2.10. Enzyme-Linked Immunosorbent Assay (ELISA)

Serum levels of pro-inflammatory cytokines, including TNF-α, interleukin (IL)-1β, and IL-6, were measured using ELISA kits (BD Biosciences, San Diego, CA, USA). All measurements were performed according to the manufacturer’s instructions. Briefly, capture antibodies (1:250) were coated into a 96-well immune plate at 4 °C overnight. The plate was washed and blocked with 3% bovine serum albumin (BSA) solution for 1 h, and the diluted samples (TNF-α, 1:10; IL-1β, 1:25; IL-6, 1:50) were placed in the wells of the plate. After 2 h, the detection antibody (1:250) was added to each well, reacted for 1 h, and then incubated with streptavidin-HRP reagent for 1 h. Absorbance was detected with substrate addition and measured at 450 nm using a microplate reader (Thermo Fisher Scientific). Cytokine levels were calculated using a standard curve.

### 2.11. Histological Analysis

Mouse kidney samples were fixed in a 4% formaldehyde solution at room temperature for 7 days and embedded in paraffin. Subsequently, the tissues were sectioned serially at 0.3 μm and stained with hematoxylin and eosin (H&E) for observation of histological alterations. Images were observed at ×200 magnification and photographed under a microscope (Olympus, Tokyo, Japan). Cell infiltration in glomerular capsule, tubular dilatation, cell death, and cast formation were scored 1 to 5 in terms of the severity of the whole cortical area of the kidney slices. The criteria for histological scores are shown in Table S1. The histological scores were scored in a blind manner.

### 2.12. RNA Sequencing

For the transcriptome analysis of kidney tissues, total RNAs were extracted using the TruSeq Stranded Total RNA LT Sample Prep Kit (Illumina, San Diego, CA, USA) according to the manufacturer’s instructions. Clean reads were obtained from raw data by removing adaptors, poly-N, and low-quality reads. For the calculation of the number of spliced reads mapped onto each gene, HISAT2 was used to align the data to a mouse reference. The expression level acquired by transcript quantification and reads per kilobase of transcript per million mapped reads was obtained from the expression profile. Differential expression analysis was conducted using the preprocess Core’R library with a false discovery rate of ≤0.05. KEGG enrichment analysis was also performed, and a heat map of differentially expressed genes (DEGs) was utilized. Gene expression ratios were visualized as log_2_ for each group.

### 2.13. Western Blot

TCMK-1 cells (2 × 10^6^ cells/well in a 6-well plate) were cultured in 2 mL of EMEM for 24 h. The cells were treated with 1000 µM of sodium thiosulfate and various OAA concentrations for 1 h and were subsequently stimulated with 20 µM of cisplatin for 6 h. For the mechanism study, the cells were treated with 10 µM of Nec-1 or 20 µM of z-VAD-FMK for 1 h and were then stimulated with 20 µM of cisplatin for 6 h. The cells were harvested and lysed in ice-cold cell lysis buffer (Cell Signaling Technology) containing phosphatase and protease inhibitor cocktail (0.5 mM PMSG/DTT and 5 μg/mL leupeptin/aprotinin) for 30 min at 4 °C. Afterward, the lysates were centrifuged at 12,000 rpm for 20 min at 4 °C, and the supernatants of cell lysates were separated. Equal amounts of protein lysates were subjected to electrophoresis on a 10% SDS-PAGE gel, and the protein bands were then transferred to a polyvinylidene difluoride membrane. After blocking with 5% BSA, the membrane was incubated with the target primary antibody, washed, and subsequently incubated with anti-IgG horseradish peroxidase-conjugated secondary antibody. The primary and secondary antibodies were used at dilutions of 1:1000 and 1:2000, respectively. Immunoreactive protein bands were visualized using a chemiluminescent substrate (Thermo Scientific), and the results were analyzed using the ChemiDoc XRS + system (Bio-Rad Laboratories). Experiments were repeated three times.

### 2.14. Quantitative Polymerase Chain Reaction (qPCR)

For nephrotoxicity assessment, the gene expression in kidney tissues and TCMK-1 cells was analyzed using qPCR. Kidney tissues were collected after sacrificing the mice and immediately frozen at −80 °C. For RNA extraction, 5 mg of kidney tissues was dissected into small pieces. TCMK-1 cells (5 × 10^5^ cells/well in a 12-well plate) were cultured in 1 mL of EMEM for 24 h. The cells were treated with 1000 µM of sodium thiosulfate and various OAA concentrations for 1 h and were subsequently stimulated with 20 µM of cisplatin for 4 h. Total RNA was isolated using the TRIzol Reagent (Invitrogen, San Diego, CA, USA) according to the manufacturer’s protocol. First-strand complementary DNA (cDNA) was synthesized using a Thermo cDNA synthesis kit (Thermo Scientific). qPCR was performed using a Bio-Rad T100 thermal cycler (Bio-Rad Laboratories) according to the manufacturer’s protocol. The primer sequences are shown in Table 1. The number of cycles was optimized to ensure that the product accumulation was in the exponential range. β-actin was used as an endogenous control for normalization. Experiments were repeated three times.

### 2.15. Statistical Analysis

Statistical analysis was performed using GraphPad Prism statistical software version7 (GraphPad Software, La Jolla, CA, USA). Treatment effects were analyzed using one-way analysis of variance, followed by Dunnett’s multiple range test. Statistical significance was set at *p* < 0.05.

## 3. Results

### 3.1. OAA Suppressed Cisplatin-Induced Apoptosis In Vitro

**Figure 1:**
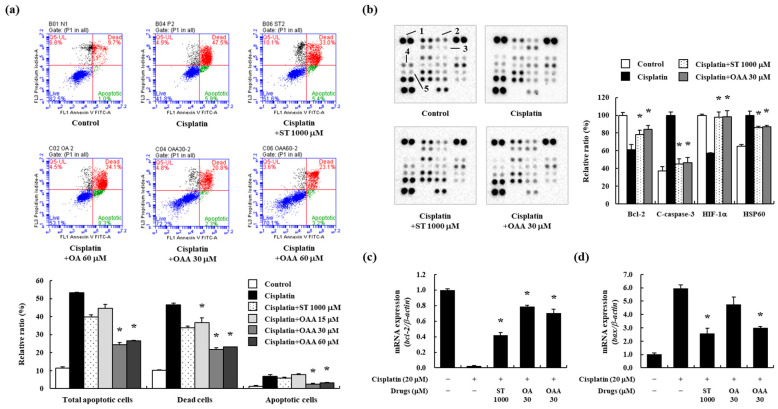
Effect of OAA on apoptotic responses in cisplatin-exposed TCMK-1 mouse kidney cells. Percentage of apoptotic cells determined using a fluorescence-activated cell sorting analysis using Annexin V and propidium iodide staining (**a**). Apoptosis-related protein expression was determined using a proteome profiler mouse apoptosis array. 1: reference; 2: Bcl-2; 3: c-caspase-3; 4: HIF-1 α; 5: HSP60 (**b**). Gene expression of Bcl-2 and Bax by qPCR (**c**,**d**). All data are presented as mean ± SD of three independent experiments. * *p* < 0.05, significantly different from cisplatin-treated group.

Considering that tubular apoptosis is one of the most adverse cellular effects of cisplatin-induced nephrotoxicity [13,15], we examined apoptosis in OAA-pretreated TCMK-1 cells. Initially, we assessed the protective effect of OAA on cisplatin-induced cell death using the WST assay. OAA pretreatment reduced cisplatin-induced cell death in a concentration-dependent manner (Figure S1a). Subsequently, TCMK-1 cells stained with Annexin V and propidium iodide were analyzed by flow cytometry to characterize cisplatin-induced cell death. Cisplatin treatment led to an increase in both early and late apoptotic cells, OAA treatment demonstrated a more significant decrease compared to OA treatment (Figure 1a). Then, a mouse apoptosis array was performed in TCMK-1 cells. Bcl-2 and hypoxia-inducible factor-α (HIF-α) proteins were decreased by cisplatin treatment, and increased by OAA treatment and, similarly, by ST treatment. Cleaved-caspase-3 and heat shock protein 60 (HSP60) were increased by cisplatin treatment and decreased by OAA treatment (Figure 1b). Gene expression of the apoptosis regulators Bcl-2 and Bax was examined in OA- and OAA-pretreated TCMK-1 cells using real-time PCR. OAA treatment resulted in decreased Bax expression and increased Bcl-2 expression (Figure 1c,d). These results suggested that cisplatin induced apoptosis in kidney cells, whereas OAA inhibited apoptosis.

### 3.2. OAA Alleviated Cisplatin-Induced Kidney Injury In Vivo

**Figure 2:**
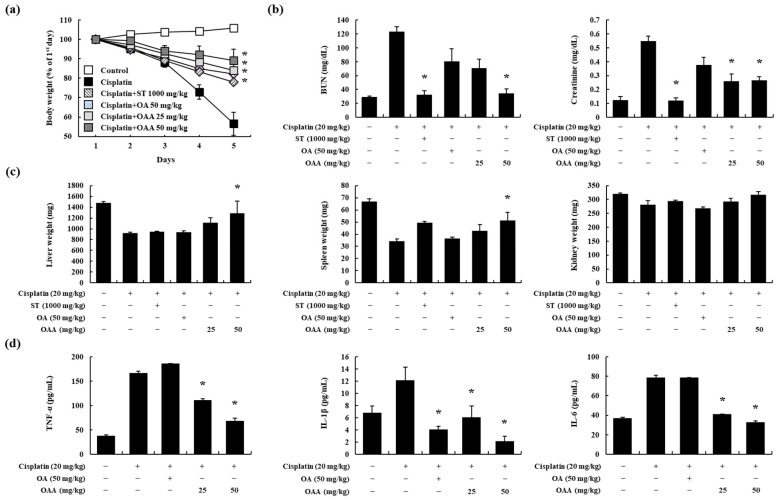
Effect of OAA on renal dysfunction in a mouse model of cisplatin-induced nephrotoxicity. Body weight change in C57BL/6 mice over 5 days in each group (**a**). Serum BUN and creatinine levels were determined (**b**). Liver, spleen, and kidney tissue weights were determined (**c**). Serum TNF-α, IL-1β, and IL-6 levels were measured using ELISA (**d**). All data are presented as mean ± SD. * *p* < 0.05, significantly different from cisplatin-treated group.

Nephrotoxicity is a recognized adverse effect associated with cisplatin treatment [1]. Therefore, we investigated the protective effects of OAA in a mouse model of cisplatin-induced kidney injury. After the oral administration of OAA, cisplatin was injected intraperitoneally at a single dose of 20 mg/kg. The cisplatin-treated group exhibited a body weight of more than 40%, and the OAA-treated group recovered more than the ST-treated group (Figure 2a). Serum BUN and creatinine levels increased in the cisplatin-treated group, whereas OAA treatment led to a significant reduction, particularly in the 50 mg/kg OAA-treated group (Figure 2b). Similar to body weight, the kidney, liver, and spleen tissue weights also decreased in the cisplatin-treated group and recovered in the group treated with 50 mg/kg OAA (Figure 2c). Additionally, serum pro-inflammatory cytokines, such as TNF-α, IL-1β, and IL-6, which exhibited elevations in the cisplatin-treated group, were significantly decreased by OAA treatment (Figure 2d).

**Figure 3:**
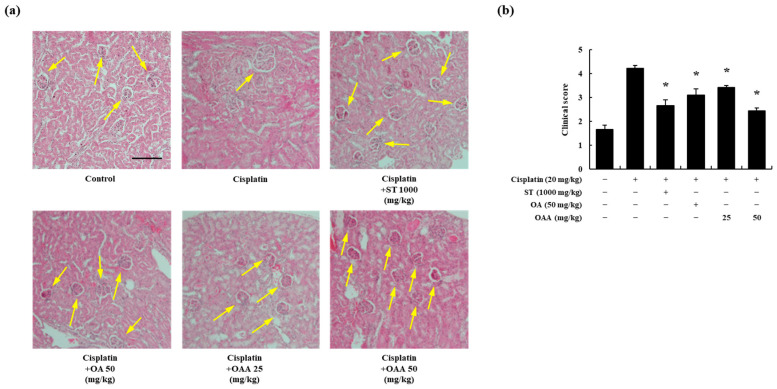
Effect of OAA on renal histology in a mouse model of cisplatin-induced nephrotoxicity. Representative histology of H&E-stained renal tissues (×200). Scale bar = 500 µm. Arrows indicate glomerular capsule (**a**). Clinical score of renal toxicity in the kidneys. Tubular cell death, tubular dilation, and cast formation were scored from 1 to 5 in terms of the severity of the kidney slices (**b**). All data are presented as mean ± SD. * *p* < 0.05, significantly different from cisplatin-treated group.

Histological changes were observed on H&E-stained kidney tissues. OAA significantly suppressed the cisplatin-induced tubular dilation and cast formation (Figure 3a,b). These findings collectively suggest that OAA mitigates cisplatin-induced kidney injury, as evidenced by improvements in body weight, organ weights, serum biochemical markers, pro-inflammatory cytokine levels, and histological features.

### 3.3. OAA Regulated the Pattern of Gene Expression through RNA Sequencing In Vivo

**Figure 4:**
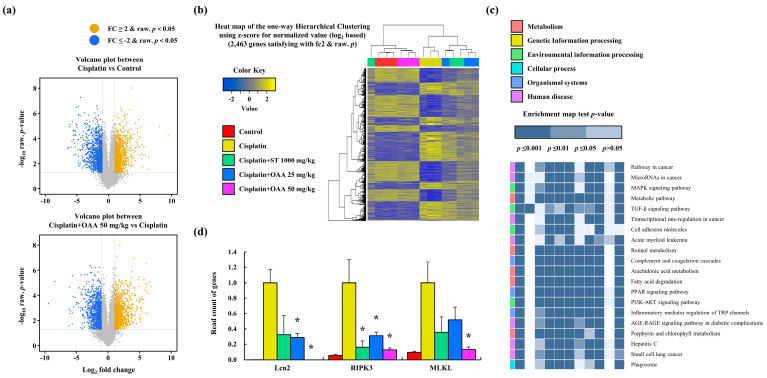
Pattern of gene expression in renal tissues in a mouse model of cisplatin-induced nephrotoxicity. The volcano plot shows the correlation between DEGs. Cisplatin vs. control and cisplatin + 50 mg/kg OAA vs. cisplatin (**a**). Cluster heatmap shows the DEGs in each group (**b**). KEGG pathway showing the top 20 enriched pathways (**c**). Read count of *lcn2* and necroptosis-related genes, such as *ripk3* and *mlkl*, in RNA sequencing (**d**). All data are presented as mean ± SD. * *p* < 0.05, significantly different from cisplatin-treated group.

RNA sequencing was performed on mouse kidney samples to identify differentially expressed genes (DEGs) and investigate the potential effects of OAA on cisplatin-induced nephrotoxicity. In each group, genetic factors that could serve as novel targets for further investigation were identified. The volcano plot indicated a total of 2463 DEGs that were significantly altered by cisplatin treatment (1184 upregulated and 1279 downregulated genes, cisplatin-treated group vs. control group) and were affected by OAA (1205 upregulated and 1079 downregulated genes, 50 mg/kg OAA treated group vs. cisplatin-treated group) (Figure 4a). A heat map was generated to visualize the DEGs in each group, which showed that the gene expression patterns in the 50 mg/kg OAA-treated and control groups were the most similar (Figure 4b). Using the KEGG pathway database, we identified DEGs related to cancer mechanisms (Figure 4c). To confirm the apoptosis pathway, we analyzed the read counts of apoptosis-related genes such as Bax, caspase7, and caspase8 in the RNA sequencing results, as depicted in Figure S3. The levels of necroptosis-related factors (RIPK3 and MLKL) increased in the cisplatin-treated group but decreased in the OAA-treated group. Furthermore, LCN2 increased significantly in the cisplatin-treated group but decreased in the OAA-treated group (Figure 4d). The results from RNA sequencing suggest that OAA not only alleviates cisplatin-induced cell death through apoptosis but also mitigates non-apoptotic cell death, specifically necroptosis. This comprehensive analysis enhances our understanding of the molecular mechanisms underlying the protective effects of OAA against cisplatin-induced nephrotoxicity.

### 3.4. OAA Inhibited Cisplatin-Induced Necroptosis

**Figure 5:**
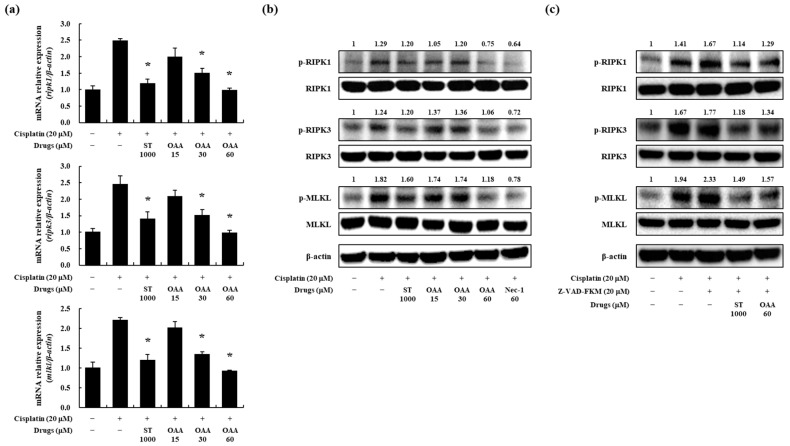
Effect of OAA on the necroptosis pathway in cisplatin-exposed TCMK-1 mouse kidney cells. The mRNA expression of necroptosis-related genes, such as *ripk1*, *ripk3*, and *mlkl*, measured by qPCR (**a**). Phosphorylation of necroptosis-associated proteins, including RIPK1, RIPK3, and MLKL, was measured by Western blot analysis (**b**). Phosphorylation of necroptosis-related proteins, such as RIPK1, RIPK3, and MLKL, was measured by Western blot analysis (**c**). All data are presented as mean ± SD of three independent experiments. * *p* < 0.05, significantly different from cisplatin-treated group.

After RNA sequencing, we evaluated necroptosis, another form of kidney cell death, in cisplatin-induced nephrotoxicity. Necroptosis induced by TNF receptors, followed by RIPK1, RIPK3, and MLKL, is considered the main factor in this pathway [27,32]. We assessed the effect of OAA on cisplatin-induced necroptosis at the mRNA level. Cisplatin treatment resulted in an upregulation of RIPK1, RIPK3, and MLKL expression in TCMK-1 cells, whereas OAA treatment effectively inhibited their expression (Figure 5a).

Given that a prior study demonstrated the inhibitory effects of Nec-1, a RIPK1 inhibitor, on necroptosis [33], we performed a Western blot analysis to confirm the effects of OAA. We utilized Nec-1 as a positive control to inhibit RIPK1 phosphorylation in TCMK-1 cells. Treatment with Nec-1 inhibited cell death induced by cisplatin treatment in TCMK-1 cells (Figure S1b). RIPK1, RIPK3, and MLKL protein expression and phosphorylation were measured after stimulation with cisplatin for 6 h. OAA, ST, and Nec-1 were treated 1 h before stimulation. Phosphorylation of these proteins was inhibited similar to Nec-1 more by OAA pretreatment than by ST pretreatment in a concentration-dependent manner (Figure 5b). 

We investigated whether OAA inhibited necroptosis via the RIPK pathway. z-VAD-FMK was pretreated to further examine the role of OAA in cisplatin-induced necroptosis. z-VAD-FMK pretreatment blocked the activation of caspase-3, thereby hindering apoptosis and enhancing necroptosis [33]. Western blot analysis revealed that z-VAD-FMK increased the phosphorylation of necroptosis-related proteins such as RIPK1, RIPK3, and MLKL. Importantly, OAA pretreatment diminished the phosphorylation of necroptosis-related proteins, which was enhanced by co-treatment with cisplatin and z-VAD-FMK (Figure 5c). The densities of the Western blot bands are presented in Figure S4. These results indicate that OAA prevented cisplatin-induced cell death, especially necroptosis, in kidney cells.

## 4. Discussion

Kidney toxicity is widely acknowledged as one of the most dose-limiting factors for cisplatin administration. In clinical practice, hydration via intravenous injection and magnesium supplementation has protective effects against cisplatin-induced kidney toxicity [43]. While various recent studies have reported that the nephrotoxicity of cisplatin could be ameliorated, FDA-approved drugs with protective adjuvants have not yet been established. Therefore, we focused on developing a new and effective adjuvant derived from natural products to reduce cisplatin-induced nephrotoxicity without diminishing anti-tumor efficacy.

OAA, a major triterpenoid compound from *V. angularis*, exhibits various pharmacological effects. In a mouse model of allergic contact dermatitis, OAA treatment reduced the levels of Th1 and Th17 cytokines. Furthermore, OAA treatment led to a decrease in the gene expression of TNF-α, IL-1β, and IL-6 in TNF-α/IFN-γ-stimulated HaCaT cells [44]. OAA administration suppressed the upregulation of pro-inflammatory cytokines in collagen-induced arthritic mouse joints [37]. Additionally, OAA decreased the expression of TNF-α in DNP-HSA-stimulated RBL-2H3 cells [45]. However, the protective effects of OAA against cisplatin-induced nephrotoxicity have not been studied. In this study, to investigate the protective effects of OAA in a mouse model of cisplatin-induced kidney injury, we provided OAA via oral administration and cisplatin via intraperitoneal injection. Compared to the cisplatin-treated group, the 50 mg/kg OAA-treated group showed a reduction in body and organ weight loss and a decrease in serum BUN, creatinine, and pro-inflammatory cytokine levels. To delve deeper into the mechanism by which OAA inhibits cisplatin-induced kidney injury, we directed our attention towards mitigating cell death, particularly apoptosis and necroptosis, induced by cisplatin treatment.

Previous studies have reported that oleanolic acid (OA) had potential anti-tumor effects in various tumor cell lines. OA treatment induced apoptosis in osteosarcoma cells, a process regulated by Bcl-2 and caspase-3 through inhibition of the Notch signaling pathway [46]. In contrast, OA had protective effects in neuronal cells, ameliorating oxidative stress and neuronal apoptosis by inhibiting the Nrf2/HO-1 pathway [47]. These studies suggest that OA has protective effects on normal cells and anti-tumor effects on cancer cells. To investigate the protective effects of OAA against cisplatin-induced kidney injury, cisplatin was treated in OAA-pretreated TCMK-1 cells. The results indicated that OA and OAA treatment reduced cell death and apoptosis, inhibited the expression of Bax and cleaved caspase-3, and increased the expression of Bcl-2 in cisplatin-treated TCMK-1 cells. Notably, OAA exhibited a stronger protective effect against cisplatin-induced kidney cell apoptosis than OA.

To identify the genetic factors as potential biomarkers or mechanisms of cisplatin-induced nephrotoxicity, we performed RNA sequencing on mouse kidney samples. Cisplatin treatment increased the expression of LCN2, an early diagnostic marker of kidney toxicity that can be used to assess its severity and complications [9]. LCN2 was significantly decreased after treatment with Nec-1, an inhibitor of necroptosis [48]. These results suggest that LCN2 expression could be increased by necroptosis and in our results, LCN2 and necroptosis-related factors were increased in the cisplatin-treated group, whereas those in the 50 mg/kg OAA-treated group were decreased. Therefore, we confirmed that cisplatin-induced necroptosis in TCMK-1 cells.

Necroptosis is recognized an anti-tumor mechanism found in various cancers, including breast and colon cancers, and in apoptosis-resistant HepG2/DDP cells [49,50,51]. However, cisplatin-induced necroptosis in the kidneys poses a serious adverse effect, prompting recent studies to identify drugs that could reduce this phenomenon [40,52]. Our in vivo results indicated that the serum TNF-α levels were increased in the cisplatin-treated group but decreased in the OAA-treated group, suggesting that OAA regulated necroptosis. As expected, OAA inhibited the gene expression of necroptosis-related factors, such as RIPK1, RIPK3, and MLKL, which were upregulated by cisplatin. Furthermore, at the protein level, cisplatin induced the phosphorylation of these factors, whereas OAA treatments inhibited the phosphorylation of these factors. Inhibition of the phosphorylation of these proteins is crucial for ameliorating cisplatin-induced kidney injury. Additionally, co-treatment with cisplatin and z-VAD-FMK increased necroptosis, whereas OAA suppressed necroptosis. OAA decreased the phosphorylation of these proteins, which was enhanced by cisplatin and z-VAD-FMK. These results suggested that OAA protected against cisplatin-induced nephrotoxicity by inhibiting necroptosis-related factors.

Finally, for future studies, it is necessary to determine the innocuous dosage of OAA through toxicity evaluation and pharmacokinetic studies. These studies are essential for the development of the drug. Combining these studies and the present results is expected to further increase the potential for OAA to develop as an anti-cancer adjuvant. 

## 5. Conclusions

In summary, our study demonstrated that OAA ameliorated cisplatin-induced apoptosis and necroptosis. Additionally, OAA suppressed cisplatin-induced kidney dysfunction and inflammation in a mouse model. This study also identified a potential clinical target for reducing the side effects of cisplatin. Taken together, OAA may serve as an anti-cancer adjuvant for reducing kidney injury.

## Figures and Tables

**Table 1:** Primer sequences for qPCR.

Gene	Origin	Forward (5′–to–3′)	Reverse (5′–to–3′)
RIPK1	Mouse	GAC TGT GTA CCC TTA CCT CCG A	CAC TGC GAT CAT TCT CGT CCT G
RIPK3	Mouse	GAA GAC ACG GCA CTC CTT GGT A	CTT GAG GCA GTA GTT CTT GGT GG
MLKL	Mouse	CTG AGG GAA CTG CTG GAT AGA G	CGA GGA AAC TGG AGC TGC TGA T
LCN2	Mouse	GGA CCA GGG CTG TCG CTA CT	GGT GGC CAC TTG CAT TGT
Bax	Mouse	TGG CAG CTG ACA TGT TTT CTG AC	TCA CCC AAC CAC CCT GGT CTT
Bcl-2	Mouse	TCG CCC TGT GGA TGA CTG A	CAG AGA CAG CCA GGA GAA ATC
β-actin	Mouse	TAG ACT TCG AGC AGG AGA TG	TTG ATC TTC ATG GTG CTA GG

## Data Availability

The data presented in this study are available on request from the corresponding author.

## Supplementary Materials

The following supporting information can be downloaded at https://www.mdpi.com/article/10.3390/toxics12040301/s1: Figure S1: Effect of OAA on cell death in cisplatin-exposed TCMK-1 cells; Figure S2: Schematic representation of the experimental design; Figure S3: Effect of OAA on apoptosis-related gene expression in a mouse model of cisplatin-induced nephrotoxicity; Figure S4: The relative density of Western blot band of cisplatin-treated TCMK-1 cells; Table S1: Criteria for histological scores.
