# Supplementary material for: Oleanolic Acid Acetate Alleviates Cisplatin-Induced Nephrotoxicity via Inhibition of Apoptosis and Necroptosis In Vitro and In Vivo
Source: Toxics. 2024 Apr 18;12(4):301. doi: 10.3390/toxics12040301 (PMC11054587; doi:10.3390/toxics12040301)
Supplement: Supplementary file 1 [file toxics-12-00301-s001.zip › toxics-2939790-supplementary.pdf]

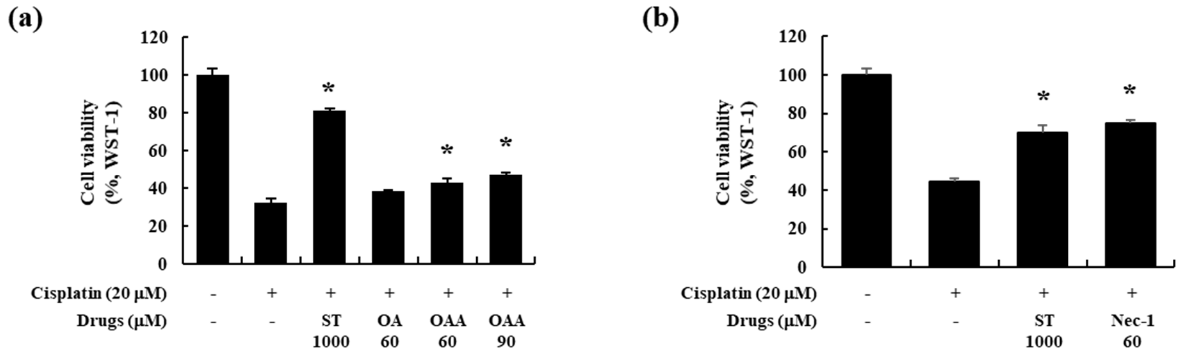

Figure S1. Effect of OAA on cell death in cisplatin-exposed TCMK-1 cells. Viability of TCMK-1 cells was analyzed using WST analysis after pretreatment with various concentrations of OAA or OA, followed by stimulation with cisplatin for 21 h (**a**). Viability of TCMK-1 cells was analyzed using WST analysis after pretreatment of Nec-1, followed by stimulation with cisplatin for 21 h (**b**). All data are presented as mean ± SD of three independent experiments. *$p < 0.05$, significantly different from cisplatin-treated group.

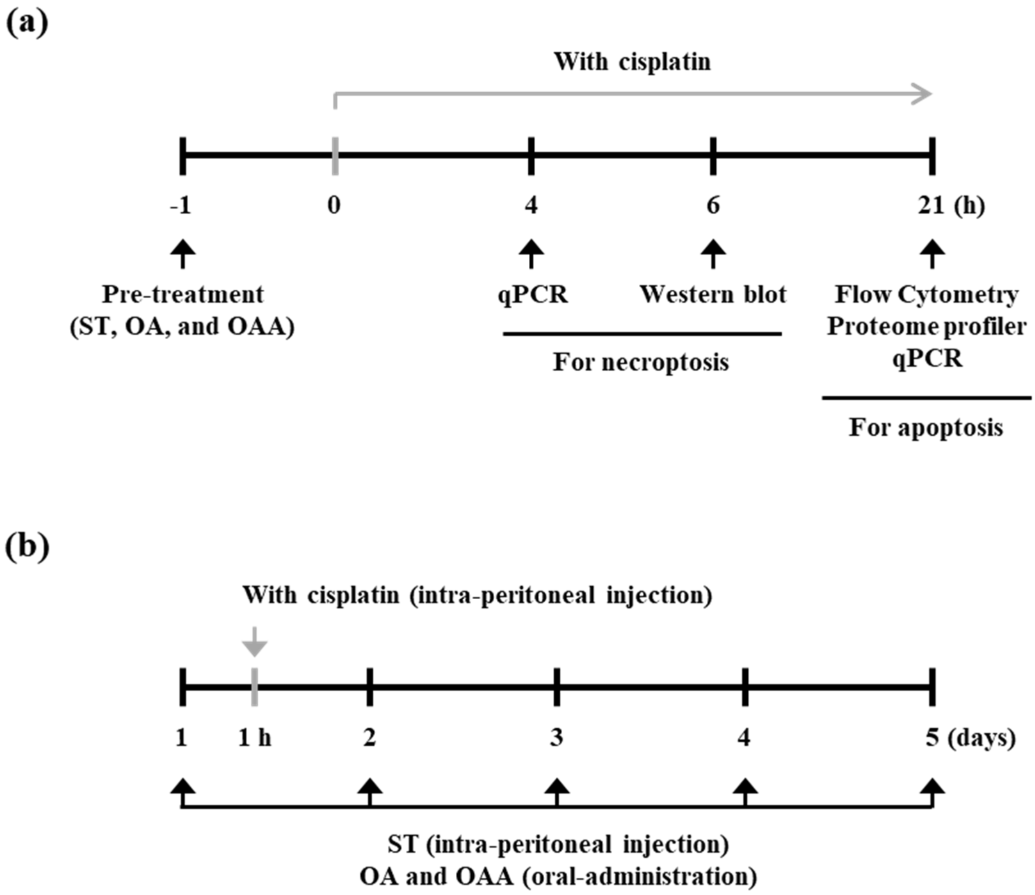

Figure S2. Schematic representation of the experimental design. TCMK-1 cells were treated with ST, OA, and OAA for 1 h and were subsequently stimulated with cisplatin. After 4 h, the cells were harvested for qPCR. After 6 h, the cells were harvested for Western blot. After 21 h, the cells were harvested for apoptosis assay (**a**). OA and OAA were orally administered once daily for 5 days. ST was intraperitoneally injected once daily for 5 days. Cisplatin was intraperitoneally injected at 1 h after drug administration on the first day (**b**).

(a)

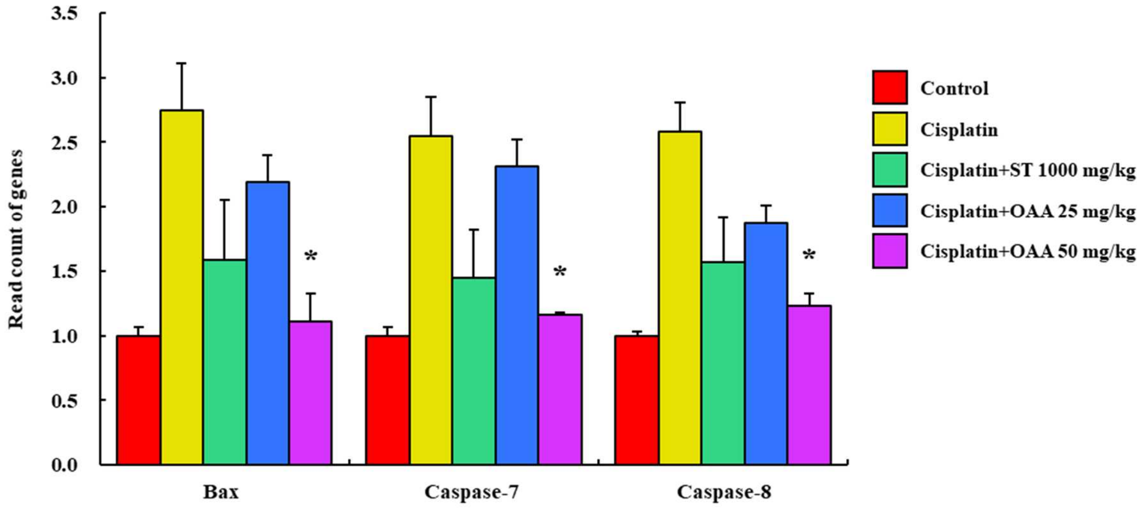

Figure S3. Effect of OAA on apoptosis-related gene expression in a mouse model of cisplatin-induced nephrotoxicity. Read count of apoptosis-related gene, such as *Bax*, *caspase7*, and *caspase8*, in RNA sequencing result. All data are presented as mean ± SD. *p < 0.05, significantly different from the cisplatin-treated group.

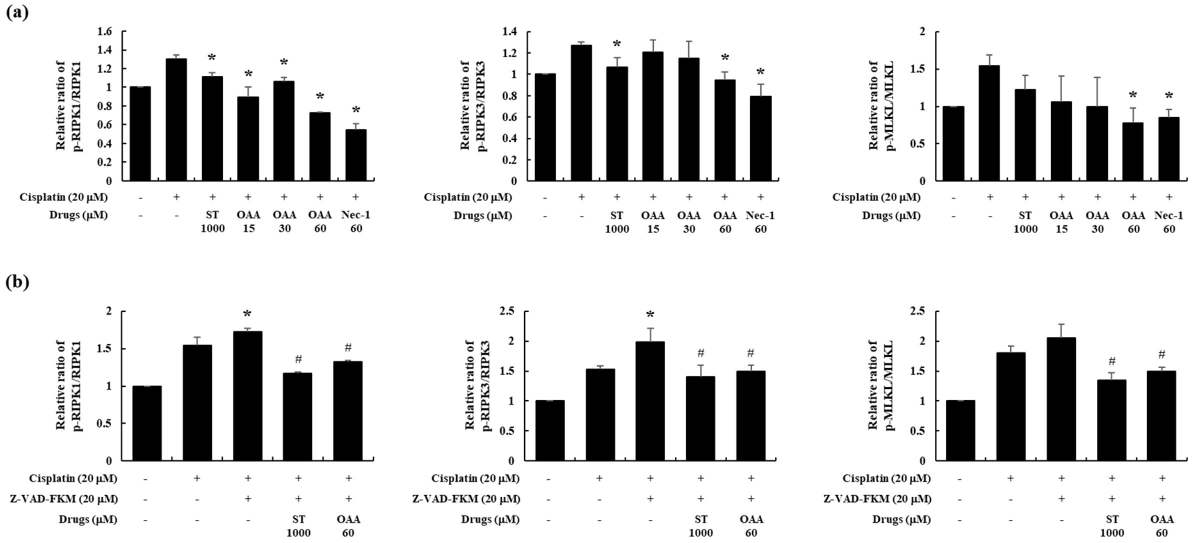

Figure S4. The relative density of the Western blot bands of cisplatin-treated TCMK-1 cells. The Western blot bands were quantified using densitometry. The related ratios of p-RIPK1/RIPK1, pRIPK3/RIPK3, and p-MLKL/MLKL are indicated in Figure 5b (a) and Figure 5c (b). All data are presented as mean ± SD of three independent experiments. *p < 0.05, significantly different from the cisplatin-treated group. #p < 0.05, significantly different from z-VAD-FMK and the cisplatin co-treated group.

Table S1. Criteria for histological scores.

Score	Grade	Overview	Glomeruli	Tubules
**1**	Normal	Normal	Normal	Normal
**2**	Mild	More or less normal aspect<br>High glomerular cell count	Apoptosis of endothelium cells<br>Inflammatory infiltrate	Little dilation<br>Normal basal membrane<br>No proteine cylinders
**3**	Moderate	Tubular dilation<br>Tubular cell damage	Same as grade 1	Apoptotic cells<br>More pronounced dilation<br>Thickened basal membrane<br>Little tubular protein cylinders<br>Regenerating cells (mimotic activity)
**4**	Pronounced	Stronger tubular dilation<br>Cell-rich infiltrate<br>Regenerating tubules	Smaller vascular lumina<br>Few erythrocytes	Flat epithelium<br>Partly, complete loss of epithelium<br>Stronger dilation<br>Inflammatory infiltrate<br>Regeneration present<br>More thickened basal membrane
**5**	Severe	Severe tubular dilation	Same as grade 3<br>More optical empty space due to glomerular shrinkage	Same as grade 3, but more empty cylinders<br>Peripheral fibrosis
